# Mining CK2 in Cancer

**DOI:** 10.1371/journal.pone.0115609

**Published:** 2014-12-26

**Authors:** Charina E. Ortega, Yoshua Seidner, Isabel Dominguez

**Affiliations:** Department of Medicine, Boston University School of Medicine, Boston, Massachusetts, 02118, United States of America; University of Torino, Italy

## Abstract

Cancer is a leading cause of death worldwide. Cancer cells proliferate uncontrollably and, many cases, spread to other parts of the body. A protein historically involved in cancer is protein kinase CK2. CK2 is a serine-threonine kinase that has been involved in cell growth, cell proliferation and cell apoptosis. CK2 functions as an oncogene when overexpressed in mouse tissues, and can synergize with known oncogenes, such as ras, to induce cell transformation in cells in culture. CK2, typically the CK2α protein, is found elevated in a number of human tumors. However, we have little information on CK2α' and CK2β proteins, and scarce information on CK2 gene transcript expression. Here, we explore the expression of CK2 transcripts in primary tumor tissues using the database Oncomine in the six cancers with the highest mortality in the U.S.A. In addition, we studied the correlation between CK2 expression and overall survival using the Kaplan-Meier Plotter database in breast, ovarian, and lung cancers. We found widespread upregulation in the expression of CK2 genes in primary tumor tissues. However, we found underexpression of CK2α' transcripts in some tumors, increased CK2β transcripts in some invasive tumors, and deregulation of CK2 transcripts in some tumor precursors. There was also correlation between CK2 expression levels and patient survival. These data provides additional evidence for CK2 as a biomarker for cancer studies and as a target for cancer therapy.

## Introduction

### Cancer prevalence

Cancer remains a leading cause of morbidity and mortality worldwide, and it is predicted to be of more importance in the next few decades. According to the World Health Organization, if global cancer rates remain unchanged, by 2030, new cancer cases will reach 21.4 million—a rise from 12.7 million new cancer cases in 2008 [1]. According to the United States Cancer Statistics, the top ten cancers by incidence rate (Age-Adjusted Invasive Cancer Incidence Rates for the 10 Primary Sites with the Highest Rates) from 2005–2011 are cancers of the prostate, breast, lung and bronchus, colorectal, corpus and uterus, urinary bladder, melanomas of the skin, non-Hodgkin lymphomas, cancers of the kidney and renal pelvis, thyroid [2]. Among them, the cancers with the highest death rates in the USA from 2005–2010 include lung and bronchus, prostate, breast, colon and rectum, ovarian and pancreatic cancers [2]. These cancers were the focus of our analysis.

### General properties and features of CK2

Protein kinase CK2 is a highly conserved serine/threonine kinase linked to diseases such as cancer [3], cardiac hypertrophy [4], multiple sclerosis [5], and inflammation [6]. Furthermore, it has been shown to be critical in embryonic development [7], and circadian rhythms [8].

CK2 regulates essential cellular processes that are characteristic to cancer development. A number of excellent publications review the role of CK2 in cancer. This include, the regulation of cell growth [9], cell proliferation [10], [11], cell survival [12], [13], cell morphology [14], cell transformation [3], signaling pathway activation [15], and angiogenesis [16], [17].

CK2 is encoded in mammals by two separate genes (CK2α and CK2α'); CK2α has broader tissue expression and higher-level expression compared to the CK2α' [18]. CK2 proteins can function as monomeric kinases or within a tetrameric complex of two CK2 kinase units and two units of the regulatory protein CK2β. In this tetrameric form, CK2β confers different substrate specificity to CK2α [19].

CK2α is an oncogene in mouse transgenic models, as CK2α overexpression in mammary gland and lymphoid compartment leads to tumor formation [20], [21]. In accordance, CK2 is overexpressed in proliferating cells [22]. In addition, there is a link between CK2 expression and environmental toxins. In animal models, CK2 is overexpressed in response to aromatic hydrocarbons [23], and is upregulated in chemically-induced liver carcinogenesis [24].

A limited number of studies testing CK2 protein and activity in human tumors found that CK2 activity and protein expression is upregulated in human cancers [25], [26] (reviewed in [22]). Although we have some knowledge on the expression of CK2α protein, we have little information on the expression of CK2α' or CK2β. Compared to protein expression levels, only a few studies test the expression levels of CK2 gene transcripts [25]. In fact, it is thought that CK2 protein and not CK2 gene transcript are elevated in cancer [22], [27]. In addition, only recently it has been shown that there is deregulated expression of the CK2α pseudogene [28], a non-protein coding CK2α sequence [29]. CK2 can serve as a prognostic marker [12], and can strengthen the multi-drug-resistant phenotype in adult cancer tissues [30], indicating the importance of studying the expression levels of CK2 genes.

**Figure 1:**
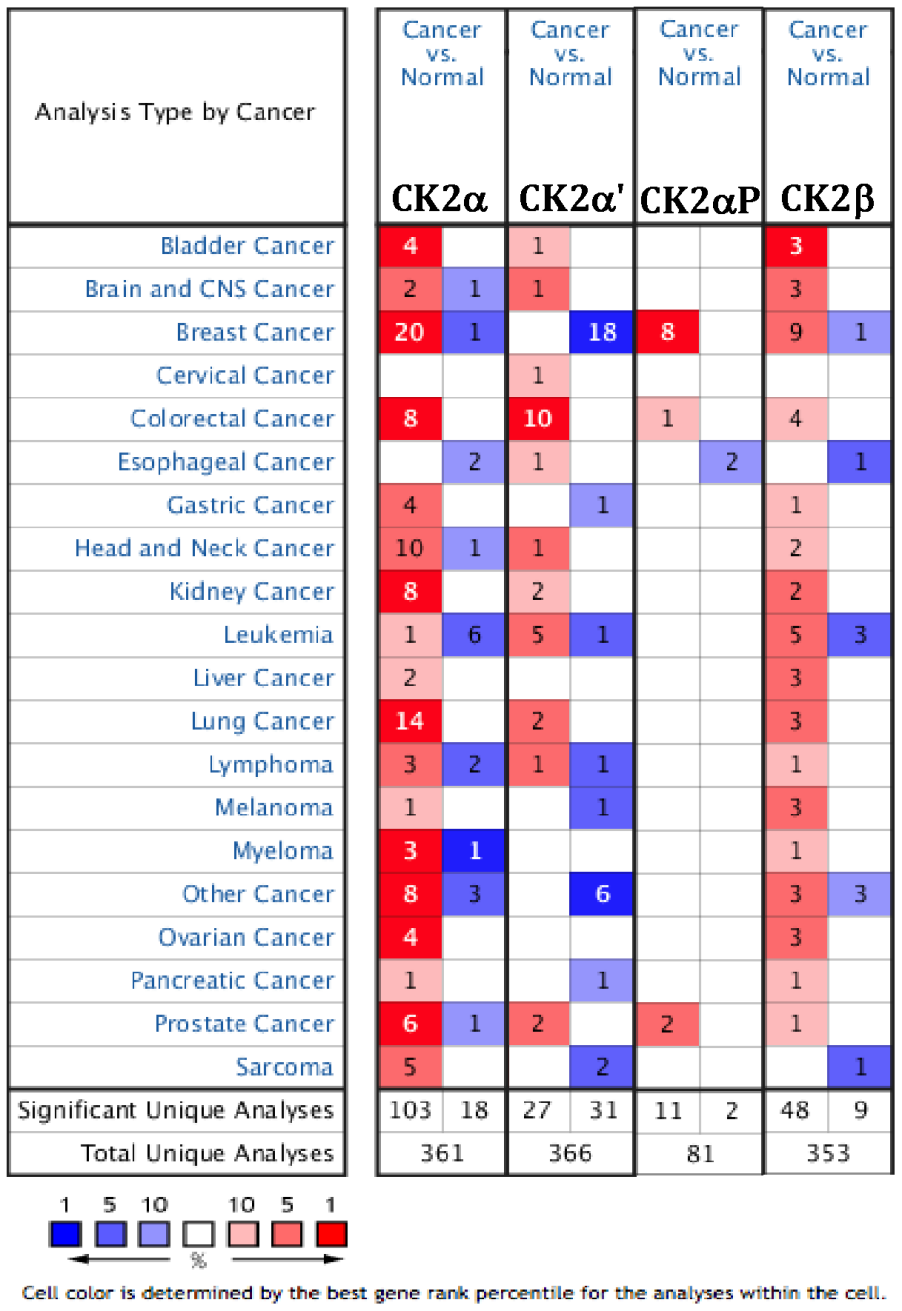
CK2 mRNA Expression in different tumor types. This graphic compares the number of datasets that had significant mRNA overexpression (left column, red) and underexpression (right column, blue) of the specified gene in cancer versus normal tissue. The datasets were obtained with the following parameters: p-value threshold of 0.01.

Therefore, we extracted data on transcript expression for CK2α, CK2α pseudogene (CK2αP), CK2α' and CK2β from the database Oncomine for lung and bronchus, prostate, breast, colon and rectum, ovarian and pancreatic cancers, focusing on clinical specimens of cancer *vs*. normal patient datasets, and separated by subtype when possible (Fig. 1). We also tested for the effect of deregulation of CK2 expression on patient overall survival using Kaplan-Meier Plotter. Here we present a summary of the results we obtained. The tables associated with the data include statistical significance, fold change, patient number and the dataset from which the information was obtained.

## Methods

### Oncomine analysis

The expression level of CK2 genes in the selected cancers was analyzed using Oncomine [31]. For this, we compared clinical specimens of cancer *vs*. normal patient datasets. In order to reduce our false discovery rate, we selected p<0.01 as a threshold. We analyzed the results for their p-values, fold change, and cancer subtype. In many instances we found several significant correlations in different tumor types, but we showed only one to three of them.

### Kaplan-Meier plotter analysis

The prognostic value of the CK2 genes in ovarian, breast and lung cancer was analyzed using Kaplan-Meier Plotter (http://kmplot.com/analysis/), a database that integrates gene expression data and clinical data [32]. To date, Kaplan-Meier Plotter contains information on 22,277 genes and their effect on survival in 2,977 breast, 1,464 ovarian and 1,715 lung cancer patients. We focused our analysis on overall survival patient information. The patient samples have been split into two groups. The two patient groups (higher and lower expression levels) were compared using a Kaplan-Meier survival plot. The hazard ratio with 95% confidence intervals and log rank p value was calculated. We analyzed the best specific probes (JetSet probes) that recognized CK2α, CK2αP, CK2α', and CK2β. In order to reduce our false discovery rate, we selected p<0.01 as a threshold.

## Results and Discussion: CK2 Transcript Expression by Cancer Type

### Lung cancer

Lung cancer has the highest mortality of all cancers in both men and women. The NCI predicts that in 2014, approximately 27% of all cancer deaths would be due to lung cancer. The NCI (http://www.cancer.gov/) [33] predicts that there would be over 224,000 new cases of lung cancer (about 14% of cancer diagnoses) in 2014, albeit a decrease in lung cancer incidence over the past few years (particularly in men). Lung cancers are divided into three groups. Non-small cell lung cancer (NSCLC) is the most common type of lung cancer (85% of lung cancers) and includes subtypes: squamous cell carcinoma, adenocarcinoma, and large cell carcinoma (LCLC). Small Cell Lung Cancer (SCLC) (10%–15% of lung cancers) quickly divides and metastasizes. Lung Carcinoid Tumors (5% of lung cancers), a type of neuroendocrine tumor, grow slowly and rarely spread. Advances in surgical and combined therapeutical approaches have increased the one-year survival by 7% (to 44% by 2005–08); however, the five-year survival rate for the combination of all stages is still low (16%); with small cell lung cancer having the lowest five-year survival rate (6%) compared to non-small cell lung cancer (18%).

#### CK2 in lung and bronchus cancer

**Figure 2:**
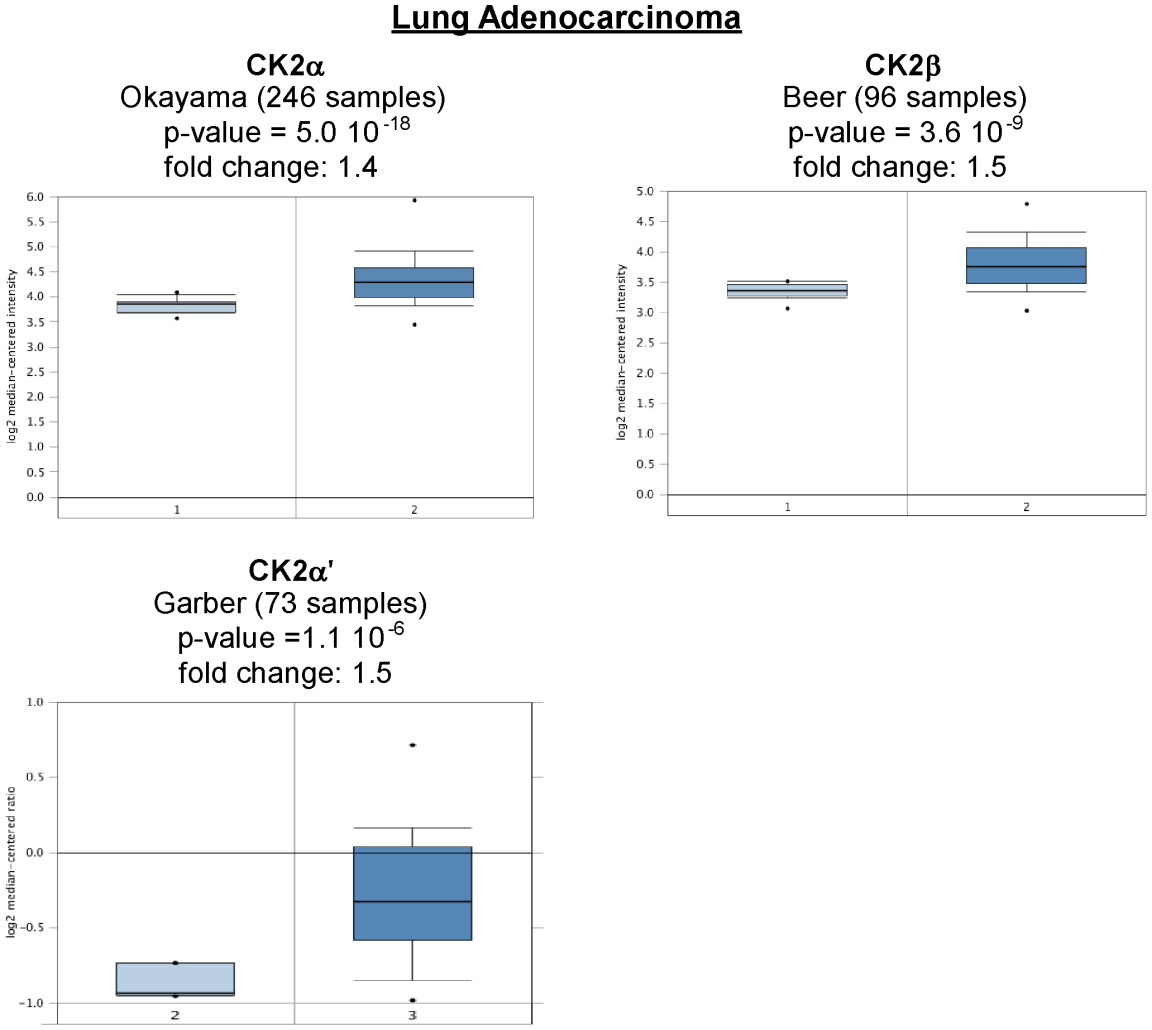
CK2 gene analysis in lung cancer (Oncomine database). Box plots derived from gene expression data in Oncomine comparing expression of a specific CK2 gene in normal (left plot) and lung cancer tissue (right plot). Only lung adenocarcinoma is shown.

Oncomine analysis of neoplastic *vs*. normal tissue showed that CK2α, CK2α', CK2β and CK2αP were significantly overexpressed in different types of lung cancer in different datasets (Table 1, Fig. 2). Overexpression of CK2α and CK2α' was found in squamous cell carcinoma and adenocarcinoma. In contrast, LCLC and SCLC had CK2α overexpression but not CK2α'. CK2β was upregulated in adenocarcinoma and LCLC but was downregulated in lung carcinoid. These data are in line with published articles on CK2 expression. For example, CK2α (*CSNK2A1*) transcript, protein and/or activity are elevated in lung cancer neoplastic tissues such as those found in squamous cell carcinomas of the lung [34], [35], [36], and in NSCLC cell lines [28], [34]. In addition, the CK2α pseudogene (*CSNK2A1P*) is amplified in non-small cell lung cancer cell lines and lung cancer tissues [28].

**Table 1:** Changes in CK2 gene expression in lung cancer.

Gene	P-Value	Fold Change	Rank (Top %)	Dataset	#Samples	Reference
**Squamous Cell Carcinoma**						
CK2α	1.5 10^−7^	18.9	1%	Bhattacharjee	203	[58]
	2.3 10^−13^	2.2	2%	Hou	156	[59]
CK2α'	5.2 10^−6^	1.8	2%	Garber	73	[60]
	0.01	2.4	10%	Bhattarcharjee	203	[58]
	9.9 10^−4^	1.5	4%	Yamagata	31	[61]
CK2αP	0.01	2.1	15%	Garber	73	[60]
**Adenocarcinoma**						
CK2α	0.001	3.3	3%	Bhattarcharjee	203	[58]
	5.0 10^−18^	1.4	1%	Okayama	246	[62]
	3.1 10^−13^	1.6	2%	Hou	156	[59]
CK2α'	1.1 10^−6^	1.5	1%	Garber	73	[60]
CK2β	3.6 10^−9^	1.5	2%	Beer	96	[63]
	8.5 10^−6^	1.6	13%	Talbot	93	[64]
**LCLC**						
CK2α	2.4 10^−9^	2.5	1%	Hou	156	[59]
CK2β	8.2 10^−5^	1.5	11%	Hou	96	[59]
**SCLC**						
CK2α	4.3 10^−4^	6	3%	Bhattarcharjee	203	[58]
**Lung Carcinoid**						
CK2α	1.2 10^−5^	6.6	3%	Bhattarcharjee	203	[58]
CK2β	0.002	−2.6	39%	Bhattarcharjee	203	[58]

Different subtypes of lung cancer are analyzed and p-values, fold changes, and datasets are included.

**Figure 3:**
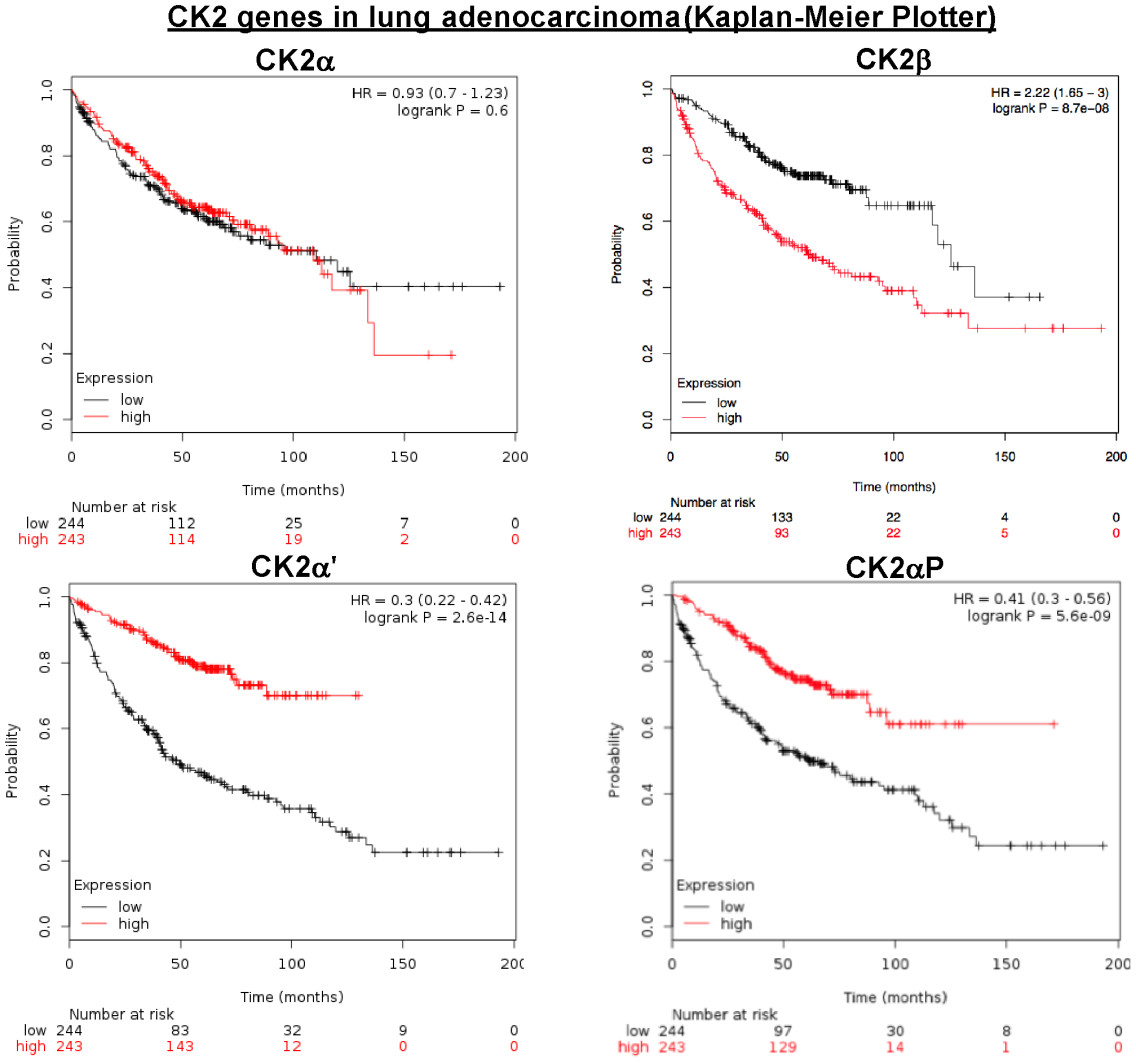
CK2 genes in lung adenocarcinoma (Kaplan-Meier Plotter). Kaplan-Meier plots showing overall survival in lung adenocarcinoma. In red: patients with expression above the median and in black, patients with expressions below the median. CK2α, p = 0.6008; CK2αP, p = 5.6 10^−9^, CK2α', p = 2.6 10^−14^; CK2β, p = 8.7 10^−8^.

Kaplan-Meier plotter analysis in overall lung cancer showed correlation between overexpression of CK2β and overall lower survival rates, and the opposite for CK2α' and CK2αP, and no significance for CK2α expression (Fig. 3). When we restricted the analysis by tumor type, no significant differences in overall survival were observed for the expression of CK2 genes in squamous cell carcinoma samples. However, in adenocarcinoma there were significant differences for CK2αP, CK2α', and CK2β mirroring the data on overall lung cancer. This indicates that the usage of CK2 gene expression as a predictor of outcome may be cancer subtype dependent (Table 1). Even though our Kaplan-Meier analysis of squamous cell carcinoma showed no significance for CK2α (p = 0.012), a published study identified CK2α (*CSNK2A1)* overexpression as one of two independent predictors of outcome (prognostic markers) in 21 patients with squamous cell carcinoma of the lung [34]. In particular, this was one of 11 genes specific of the poor prognosis cluster group (cluster II) of non-small cell lung cancers that had corresponding chromosomal aberrations. The authors validated these results independently in an additional set of 45 patients.

### Breast cancer

Excluding cancers of the skin, breast cancer is the most frequently diagnosed cancer in women. According to the NCI, an estimated 40,000 breast cancer deaths in both women and men are expected in 2014 (6.8% of all cancers) [33]. Breast cancer ranks second as a cause of cancer death in women (after lung cancer). An estimated 232,670 new cases of breast cancer are expected to be diagnosed in women in the US during 2014 (14% of all cancers). Breast cancer can happen in the ducts, the lobules and in the stroma. The most common breast cancers are ductal carcinoma (70% of cases) and lobular carcinoma (10% of cases). These can be subdivided into non-invasive (*in situ*) and invasive. Mucinous breast carcinoma is a subtype of ductal carcinoma (2–5% incidence) Breast cancer treatment can involve surgery, radiation therapy, chemotherapy, hormone therapy and/or targeted therapy. The overall 5-year survival rate is 89.2% in localized or low-grade breast cancer. Early detection and novel treatments have increased the survival rate for localized breast cancer to 98.6% and for regional breast cancer to 84.4%.

#### CK2 in Breast Cancer

**Figure 4:**
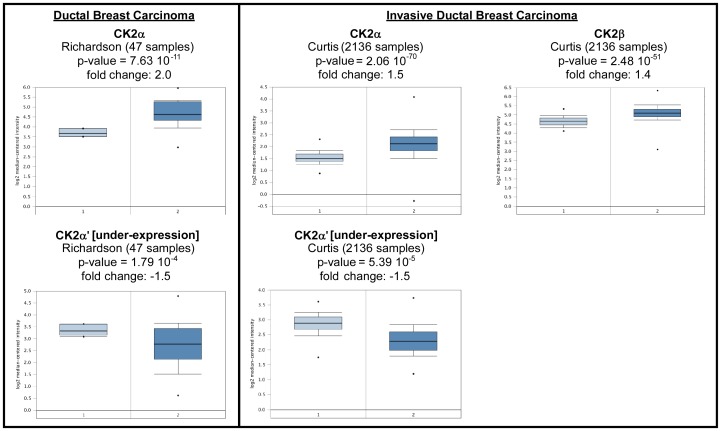
CK2 gene analysis in breast cancer (Oncomine database). Box plots derived from gene expression data in Oncomine comparing expression of a specific CK2 gene in normal (left plot) and lung cancer tissue (right plot). Invasive and non-invasive ductal breast carcinomas are featured in the box plots.

Overall, Oncomine analysis showed significant levels of both CK2α and CK2β overexpression in most breast cancer types, but strong CK2α' underexpression in all breast cancer types (Table 2, Fig. 4). A difference was found between ductal and lobular breast cancers, where ductal, but not lobular, expressed high levels of CK2α. A further difference was found in between non-invasive and invasive breast cancers. Both showed CK2α' underexpression, but in invasive breast cancer, CK2β was also very significantly overexpressed. Since CK2β protein is stabilized post-translationally by CK2α [9], increased CK2β transcript expression in CK2α overexpressing cancers, may contribute to further increase CK2β protein expression.

**Table 2:** Changes in CK2 gene expression in breast cancer.

Gene	P-Value	Fold Change	Rank (Top %)	Dataset	#Samples	Reference
**Ductal Breast Carcinoma**						
CK2α	7.43 10^−11^	2	1%	Richardson 2	47	[65]
CK2α'	2.05 10^−4^	−1.6	3%	Sorlie	85	[66]
	9.15 10^−4^	−1.6	6%	Sorlie Breast 2	167	[67]
	1.79 10^−4^	−1.5	10%	Richardson 2	47	[65]
**Invasive Ductal Breast Carcinoma**						
CK2α	8.09 10^−28^	1.6	4%	TCGA Breast	593	[68]
	2.06 10^−70^	1.5	4%	Curtis	2136	[69]
CK2α'	4.04 10^−20^	−1.5	10%	TCGA Breast	593	[68]
	5.39 10^−50^	−1.5	5%	Curtis	2136	[69]
	1.08 10^−4^	−1.6	4%	Zhao	64	[70]
CK2β	2.48 10^−51^	1.4	8%	Curtis	2136	[69]
**Lobular Breast Carcinoma**						
CK2α'	6.37 10^−4^	−1.5	4%	Zhao	64	[70]
**Invasive Lobular Breast Carcinoma**						
CK2α'	3.75 10^−11^	−1.6	5%	TCGA Breast	593	[68]
	4.51 10^−43^	−1.6	3%	Curtis	2136	[69]
CK2β	8.00 10^−39^	1.4	2%	Curtis	2136	[69]
**Mucinous Breast Carcinoma**						
CK2α	4.33 10^−5^	1.5	2%	TCGA Breast	593	[68]
	1.39 10^−16^	1.4	3%	Curtis	2136	[69]
**Invasive Mucinous Breast Carcinoma**						
CK2β	2.48 10^−51^	1.4	8%	Curtis	2136	[69]
**Breast Carcinoma**						
CK2α'	3.09 10^−5^	−1.7	8%	Curtis	2136	[69]
**Invasive Breast Carcinoma**						
CK2α	3.58 10^−15^	1.5	6%	TCGA Breast	593	[68]
**Invasive Breast Carcinoma Stroma**						
CK2α	1.2 10^−28^	−2.2	2%	Finak Breast	59	[71]
CK2α'	7.45 10^−12^	−6.8	15%	Finak Breast	59	[71]
CK2β	3.02 10^−20^	−5.7	7%	Finak Breast	59	[71]
**Tubular Breast Carcinoma**						
CK2α'	3.22 10^−36^	−1.8	2%	Curtis	2136	[69]
**Invasive Medullary Carcinoma**						
CK2αP	1.2 10^−9^	1.5	5%	Curtis	2136	[69]
CK2β	1.4 10^−10^	1.6	4%	Curtis	2136	[69]

Different subtypes of breast cancer are analyzed and p-values, fold changes, and datasets are included.

**Figure 5:**
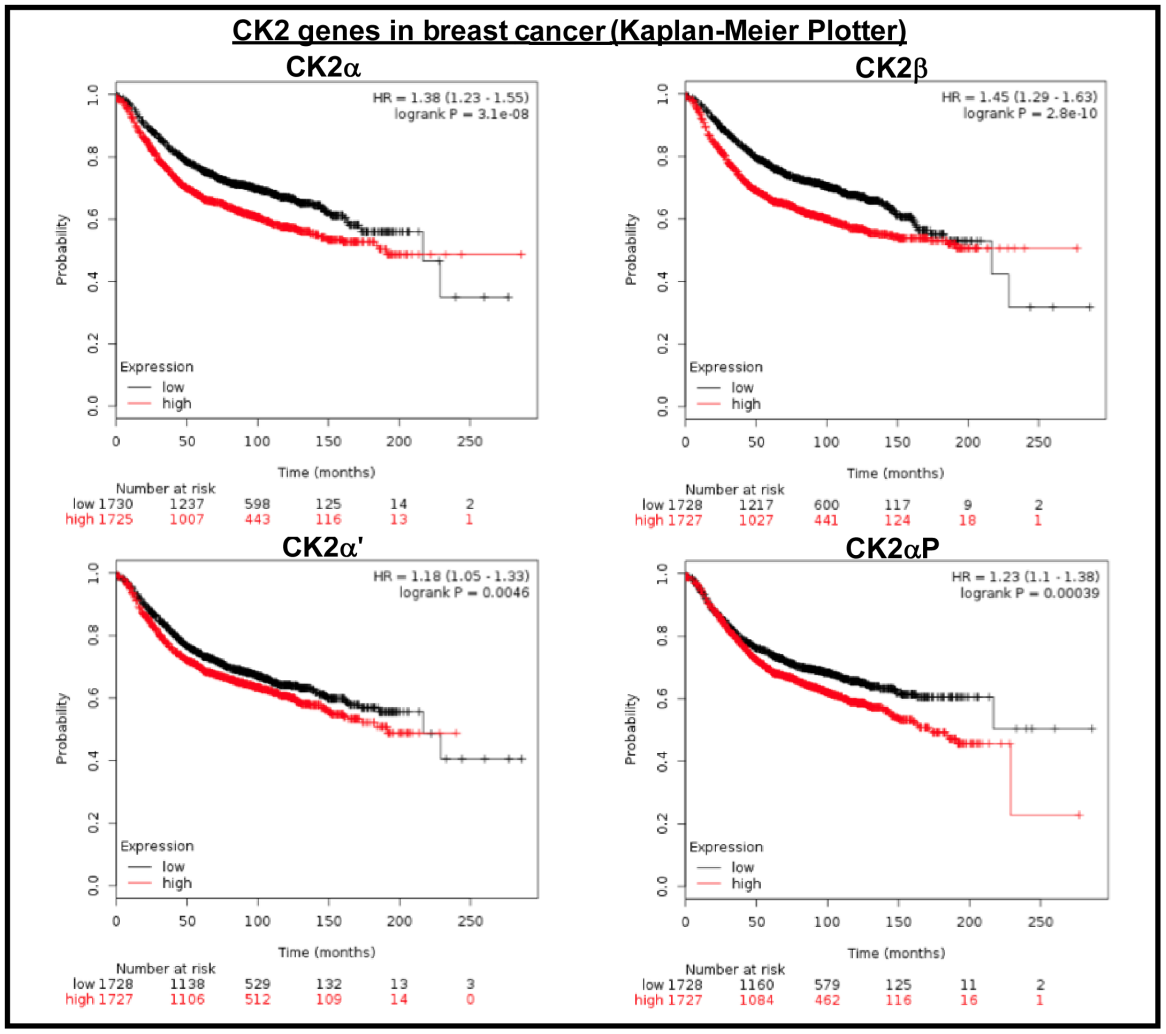
CK2 genes in breast cancer (Kaplan-Meier Plotter). Kaplan-Meier plots showing overall survival in lung adenocarcinoma. In red: patients with expression above the median and in black, patients with expressions below the median. CK2α, p = 3.1 10^−8^; CK2αP, p = 3.9 10^−4^; CK2α', p = 4.6 10^−3^; CK2β, p = 2.8 10^−10^.

Kaplan-Meier analysis revealed that in overall breast cancer high levels of expression of CK2α and CK2β correlate with lower survival rates (Fig. 5). CK2α' expression had no significant influence in survival in all breast cancers (Fig. 5). This is in line with a published study, CK2α (*CSNK2A1)* was found as part of a 186-gene “invasiveness” gene signature in breast cancer. This “invasiveness” gene signature was associated with overall survival and metastasis-free survival in patients with breast cancer, and when combined with the NIH prognostic criteria, the “invasiveness” gene signature was used to stratify patients with high-risk early breast cancer. This “invasiveness” gene signature was associated with overall survival and metastasis-free survival in patients with medulloblastoma, lung cancer and prostate cancer [37]. In addition, it has been found that unbalanced expression of CK2 genes is able to promote epithelial to mesenchymal transition in cell lines [38]. These studies emphasize the importance of analysis of the expression of CK2 genes during cancer progression.

### Colon cancer

Colon cancer is the third leading cause of cancer related deaths when men and women are considered separately, and the second leading cause when both considering both sexes. The NCI predicts that in 2014 there would be an estimated 50,310 deaths in men and women, which would be 8.6% of all cancer deaths [33]. They have estimated 136,830 new cases of colorectal cancer in 2014. When combined, colorectal cancers make up 8.2% of all new cancer cases. The most common type of colorectal cancers is adenocarcinoma (95%). Its precursor, adenomatous polyps (adenomas) are polyps that are considered to be a pre-cancerous condition. Treatments of this cancer type include surgery, chemotherapy, biological therapy, and radiation therapy. Early detection of colorectal cancers can increase survival rate when comparing localized colorectal cancers (90.3%) and regional colorectal cancers (70.4%). The relative 1-year relative survival rate is 84% and the relative 5-year survival rate for colorectal cancer is 64.9%.

#### CK2 in Colon cancer

**Figure 6:**
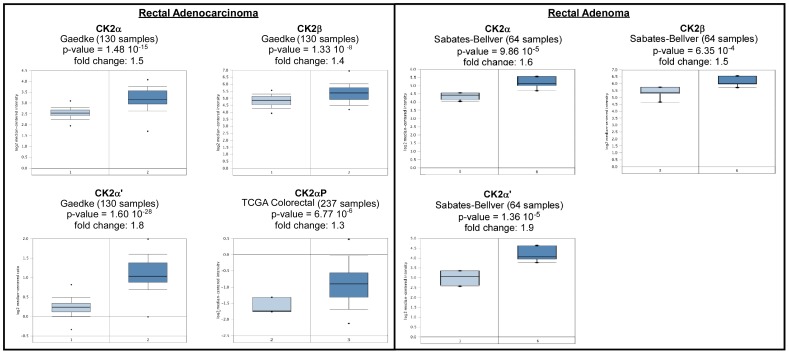
CK2 gene analysis in colorectal cancer (Oncomine database). Box plots derived from gene expression data in Oncomine comparing expression of specific CK2 genes in in normal plot (left plot) and in colorectal cancer tissue (right plot). Oncomine box plots were retrieved from rectal adenomas and adenocarcinomas.

Oncomine analysis revealed many cases of overexpression for all three CK2 genes and the CK2α pseudogene in adenocarcinomas (Table 3, Fig. 6). We found a statistically significant increase in CK2α in colon adenocarcinoma. In agreement with this finding, a published study shows that expression levels of CK2α transcripts are elevated in 10 pairs of colorectal tumor tissues compared to non-tumor tissues [39]. Oncomine analysis found that there is also overexpression of CK2 genes in adenomas (Table 4). This is relevant, because CK2 has been shown to regulate Wnt/β-catenin signaling [7]. Wnt/β-catenin signaling is major player in colorectal cancer progression as it is proposed to be the first hit to be deregulated in colon cancer progression [15].

**Table 3:** Changes in CK2 gene expression in colon cancer.

Gene	P-Value	Fold Change	Rank (Top %)	Dataset	#Samples	Reference
**Colon Carcinoma**						
CK2α	8.38 10^−7^	2.0	8%	Skrzypczak	40	[72]
CK2α'	6.63 10^−10^	2.3	2%	Skrzypczak Colorectal 2	40	[72]
	6.17 10^−15^	1.5	6%	TCGA Colorectal	237	[68]
	2.28 10^−19^	2.6	1%	Hong Colorectal	82	[73]
CK2αP	4.12 10^−9^	1.5	15%	TCGA Colorectal	237	[68]
CK2β	2.30 10^−10^	1.4	6%	Ki Colon	123	[74]
	2.71 10^−7^	1.9	7%	Skrzypczak Colorectal 2	40	[72]
**Rectal Adenocarcinoma**						
CK2α	1.48 10^−15^	1.5	9%	Gaedke	130	[75]
CK2α'	1.60 10^−28^	1.8	2%	Gaedke	130	[75]
CK2αP	6.77 10^−6^	1.3	22%	TCGA Colorectal	237	[68]
CK2β	1.33 10^−8^	1.4	20%	Gaedke	130	[75]
**Cecum Adenocarcinoma**						
CK2α	4.29 10^−7^	1.7	12%	TCGA Colorectal	237	[68]
CK2α'	8.74 10^−8^	1.5	10%	TCGA Colorectal	237	[68]
CK2αP	2.48 10^−6^	1.7	14%	TCGA Colorectal	237	[68]
CK2β	2.95 10^−5^	1.4	19%	TCGA Colorectal	237	[68]

Different subtypes of colon cancer are analyzed and p-values, fold changes, and datasets are included.

**Table 4:** Changes in CK2 gene expression in non-neoplastic colon adenomas.

Gene	P-Value	Fold Change	Rank (Top %)	Dataset	#Samples	Reference
**Colon Adenoma**						
CK2α	2.32 10^−5^	2.4	7%	Skrzypczak	40	[72]
CK2α'	2.43 10^−10^	1.6	8%	Sabates-Bellver	64	[76]
CK2β	1.31 10^−7^	1.4	14%	Sabates-Bellver	64	[76]
**Rectal Adenoma**						
CK2α	9.86 10^−5^	1.6	11%	Sabates-Bellver	64	[76]
CK2α'	1.36 10^−5^	1.9	6%	Sabates-Bellver	64	[76]
CK2β	6.35 10^−4^	1.5	18%	Sabates-Bellver	64	[76]

Different subtypes of colon adenomas are analyzed and p-values, fold changes, and datasets are included.

### Ovarian cancer

Ovarian cancer ranks fifth in cancer death among women (3% of all cancers among women), accounting for more deaths than any other cancer of the female reproductive system. According to the NCI in 2014, there would be an estimated 14,270 deaths due to ovarian cancer, accounting for 2.4% of all cancer deaths and 7.5% of cancer deaths among women [33]. There would be approximately 21,980 new cases of ovarian cancer in the USA in 2014, accounting for 1.3% of all new cancer cases. The most common type of ovarian cancer is epithelial ovarian carcinoma, which accounts for 85% to 90% of all ovarian cancers. Epithelial ovarian cancer is divided into other subtypes such as serous, which accounts for 80–85%, endometrioid, which accounts for ∼10%, and clear cell, which accounts for ∼5% of all epithelial ovarian cancers. The less-common types of ovarian cancers include germ cell tumors (less than 2% of ovarian cancers), and stromal tumors (1% of ovarian cancers). The treatment methods of ovarian cancer include surgery, chemotherapy and radiation. A woman's risk of developing ovarian cancer in her lifetime is 1 in 72, and the age at which she is diagnosed affects survival rate. 56% of women younger than 65 years survive 5 years following diagnosis, while 27% of women older than age 65 survive 5 years following diagnosis. Overall, the 1-year relative survival rate of all ovarian cancer patients is 75% and the 5-year relative survival rate is 44%. If the tumor is localized, there is a 91.9% 5-year relative survival rate, but if the tumor is regional, the survival rate drops to 72.0%.

#### CK2 in Ovarian cancer

**Figure 7:**
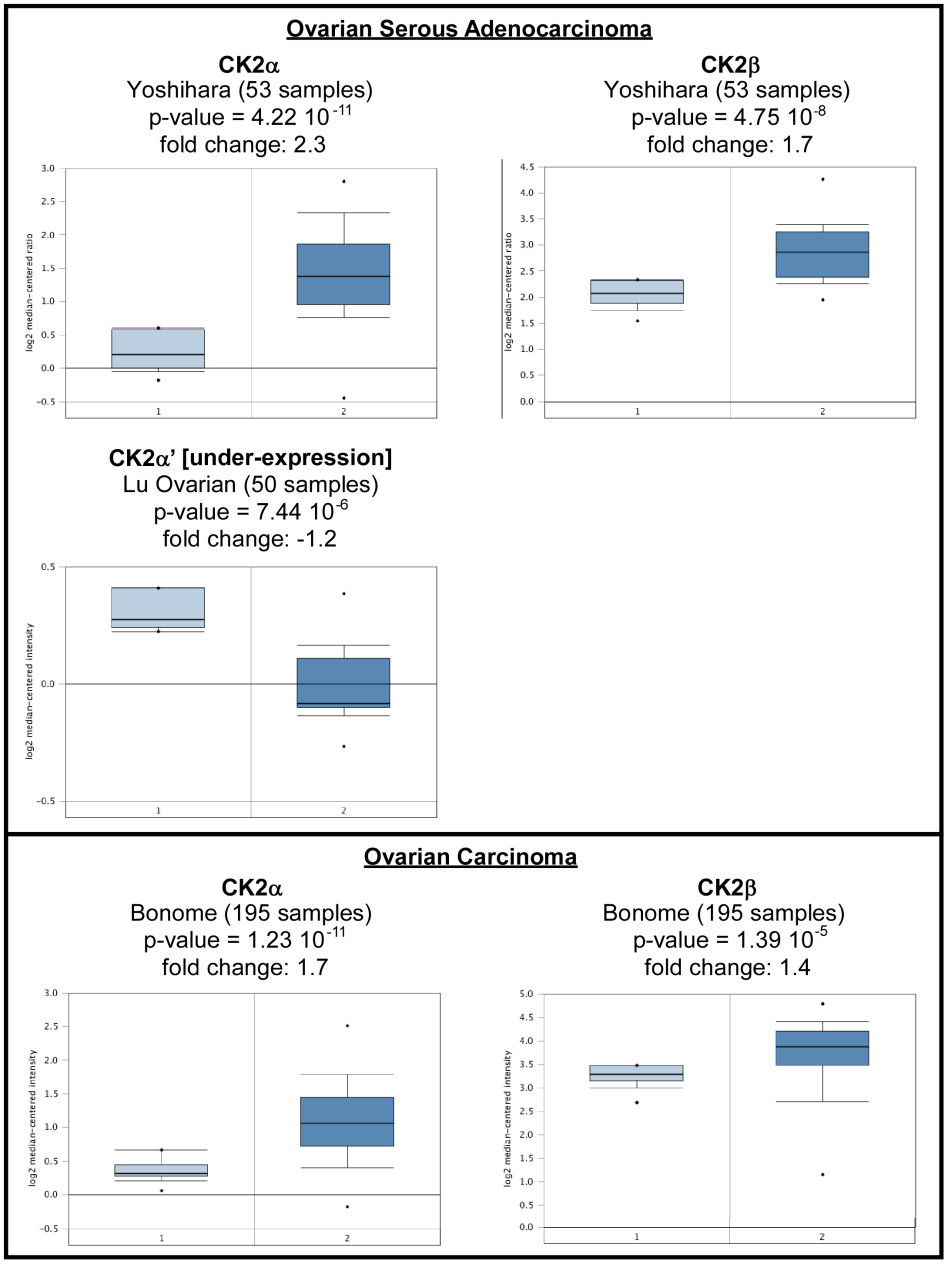
CK2 gene analysis in ovarian cancer (Oncomine database). Box plots derived from gene expression data in Oncomine comparing expression of a specific CK2 gene in normal (left plot) and lung cancer tissue (right plot). Oncomine box plots were retrieved from serous ovarian adenocarcinoma and ovarian carcinoma.

Oncomine analysis of neoplastic *vs*. normal tissue showed that CK2α and CK2β were significantly upregulated in several ovarian cancer types. CK2α' was found significantly downregulated in two ovarian tumor types. There were no significant results for CK2αP (Table 5, Fig. 7).

**Table 5:** Changes in CK2 gene expression in ovarian cancer.

Gene	P-Value	Fold Change	Rank (Top %)	DataSet	#Samples	Reference
**Ovarian Serous Adenocarcinoma**						
CK2α	4.22 10^−11^	2.3	1%	Yoshihara	53	[77]
CK2α'	7.44 10^−6^	−1.2	1%	Lu Ovarian	50	[78]
	0.005	−1.4	6%	Adib	16	[79]
CK2β	4.75 10^−8^	1.7	3%	Yoshihara	53	[77]
**Ovarian Carcinoma**						
CK2α	1.23 10^−11^	1.7	3%	Bonome	195	[80]
CK2β	1.39 10^−5^	1.4	19%	Bonome	195	[80]
**Ovarian Serous Cystadenocarcinoma**						
CK2α	6.51 10^−6^	1.7	7%	TCGA Ovarian	594	[68]
**Ovarian Endometroid Adenocarcinoma**						
CK2β	0.01	1.6	11%	Lu Ovarian	50	[78]
**Ovarian Mucinous Adenocarcinoma**						
CK2α'	1.82 10^−4^	−1.2	1%	Lu Ovarian	50	[78]

Different subtypes of ovarian cancer are analyzed and p-values, fold changes, and datasets are included.

**Figure 8:**
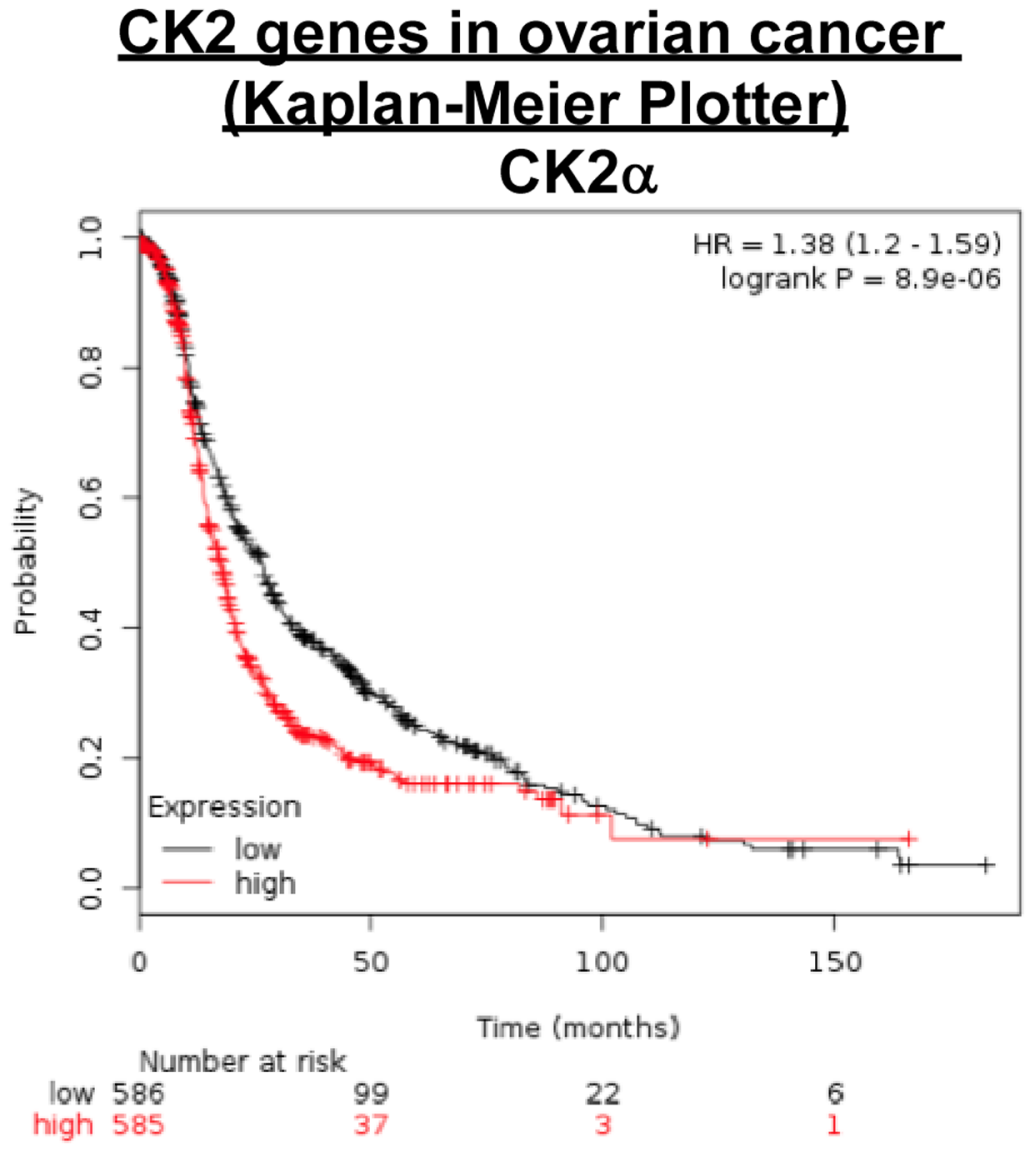
CK2 genes in ovarian cancer (Kaplan-Meier Plotter). Kaplan-Meier plots showing overall survival in ovarian cancer. In red: patients with expression above the median and in black, patients with expressions below the median. The CK2 genes and corresponding p-values are listed. CK2α, p = 8.9 10^−6^; CK2αP, p = 0.034; CK2α', p = 0.45; CK2β, p = 0.12.

Kaplan-Meier plotter analysis revealed that high-levels of expression of CK2α correlated with lower patients survival rates. In contrast, although CK2α' was significantly downregulated in some adenocarcinomas and CK2β that was upregulated in a few subtypes, Kaplan-Meier plotter analysis did not show any significance of CK2α' and CK2β expression levels in overall survival (Fig. 8).

### Prostate cancer

Prostate Cancer is the most common cancer among men, after skin cancer, according to the NCI. The NCI predicts that in 2014, there would be 233,000 new cases of prostate cancer, accounting for 14% of all new cancer cases [33]. The NCI predicts 29,480 deaths in 2014, which would account for 5% of all cancer deaths. The most common type of prostate cancer is adenocarcinoma, which accounts for about 90% of all prostate cancers. There are subtypes of adenocarcinoma including atrophic, foamy, colloid, and signet ring carcinoma. The remaining 10% of all prostate cancers include transitional cell cancer, squamous cell cancer, carcinoid, small cell cancer, and sarcomas and sarcomatoid cancers. Treatments for prostate cancer include surgery, and different therapies such as radiation, hormone, chemotherapy, chemoradiotherapy, and immunotherapy. There is an extremely high survival rate overall. When caught early and the tumor is either localized or regional, there is a 100.0% chance or a 5-year relative survival. Overall, the relative 5-year relative survival rate for localized or low-grade cancer is 99.2%.

#### CK2 in Prostate cancer

**Figure 9:**
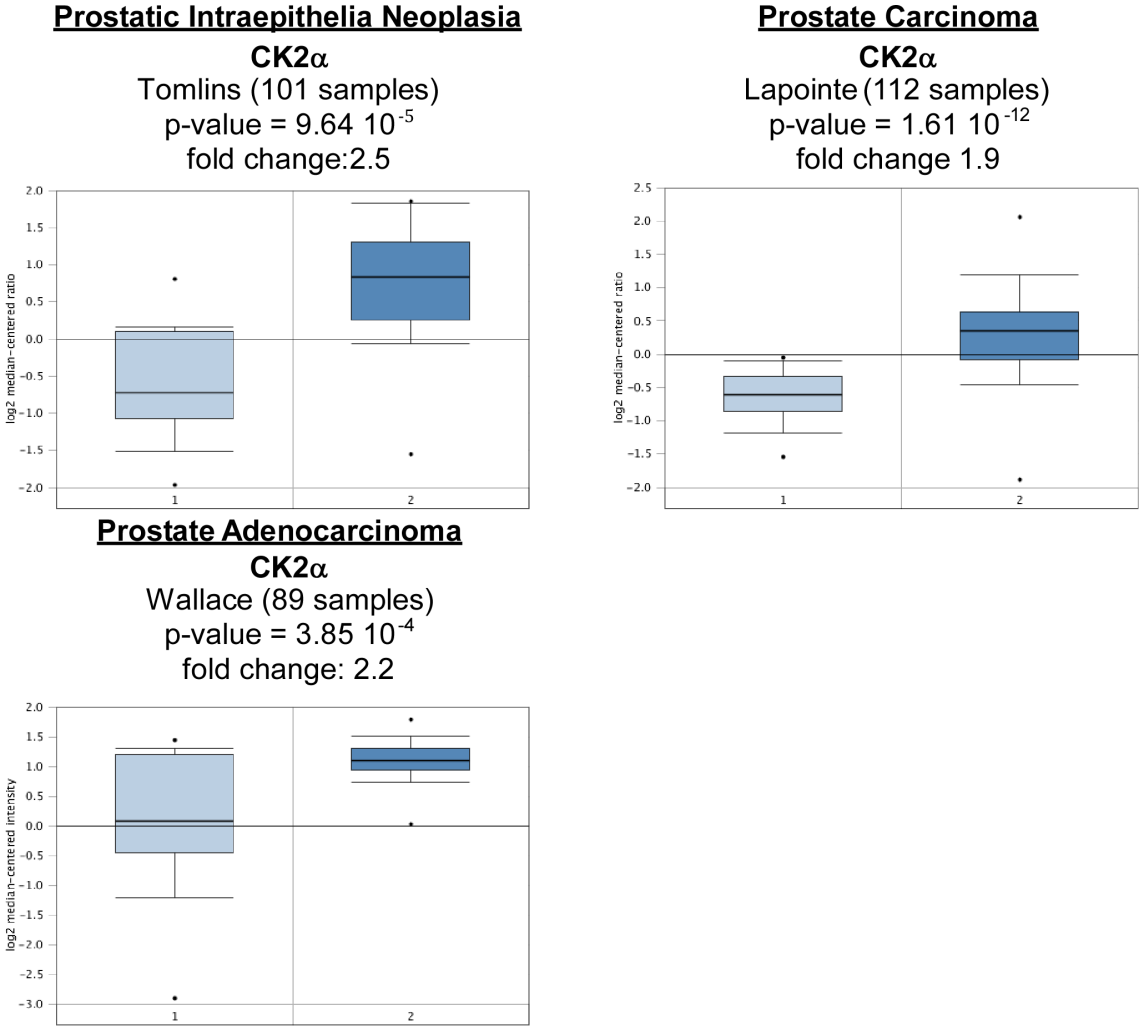
CK2α gene analysis in prostate cancer (Oncomine database). Box plots derived from gene expression data in Oncomine comparing expression of CK2α gene in normal (left plot) and various types of prostate cancer tissue (right plot). Oncomine box plots were retrieved from various types of prostate cancer.

In prostate cancer, Oncomine analysis revealed significant overexpression of CK2α in three subtypes, while CK2α' and CK2β were found only upregulated in one cancer subtype each. There were no significant results for CK2αP (Table 6, Fig. 9). This data are in line with publications showing significant increase in CK2α expression in prostate tumor lesions (p = 0.0224) [40], CK2 activity correlating with Gleason grade [41], and nuclear localization of CK2α associating with poor prognosis [42].

**Table 6:** Changes in CK2 gene expression in prostate cancer.

Gene	P-Value	Fold Change	Rank (Top %)	Dataset	#Samples	Reference
**Prostatic Intraepithelia Neoplasia**						
CK2α	9.64 10^−5^	2.5	3%	Tomlins	101	[81]
CK2α'	4.4 10^−4^	2	5%	Tomlins	101	[81]
**Prostate Adenocarcinoma**						
CK2α	3.85 10^−4^	2.2	4%	Wallace	89	[82]
**Prostate Carcinoma**						
CK2α	1.61 10^−12^	1.9	1%	Lapointe	112	[83]
	3.42 10^−5^	1.9	4%	Tomlins	101	[81]
	2.57 10^−4^	1.9	5%	Singh	102	[84]
CK2β	2.54 10^−4^	1.3	8%	Welsh	34	[85]

Different subtypes of prostate cancer are analyzed and p-values, fold changes, and datasets are included.

### Pancreatic cancer

Pancreatic Cancer is the fourth-leading cause of death from cancer in the United States, after lung cancer, colon cancer, and breast cancer, but it is the 12^th^ most common type of cancer in the United States. The NCI estimates that there would be an estimated 39,590 deaths due to pancreatic cancer in 2014, which accounts for 6.8% of all cancer deaths [33]. They estimated that there would be 46,420 new cases, making up 2.8% of all new cancer cases. There are two types of pancreatic cancer cells, pancreatic exocrine tumors and pancreatic neuroendocrine tumors. About 95% of pancreatic cancers are classified as exocrine tumors, which has many different subtypes such as adenocarcinoma, and acinar cell carcinoma. Adenocarcinoma accounts for about 90% of all pancreatic cancers, and the other subtypes are considered to be rare. Pancreatic neuroendocrine tumors account for less than 5% of pancreatic tumors, and have much better survival than pancreatic adenocarcinoma. Treatments for people with adenocarcinomas include surgery, chemotherapy, targeted therapy, and radiation therapy. Despite these treatments, the relative survival rate is fairly low. Even when the tumor is localized or regional, patients have a relatively low 5-year relative survival rate, which is 24.1% and 9.0% respectively. Accounting for all types of pancreatic cancer, there is a 6.0% that a patient can survive 5 years.

#### CK2 in Pancreatic cancer

Oncomine analysis revealed that only CK2β was significantly upregulated in pancreatic adenocarcinoma. CK2β was downregulated in pancreatic adenocarcinoma while CK2α' was downregulated in pancreatic ductal adenocarcinoma (Table 7). CK2α was overexpressed in both types of pancreatic cancer but not significantly (p-values around 0.02 to 0.03). CK2αP was not significantly changed.

**Table 7:** Changes in CK2 gene expression in pancreatic cancer.

Gene	P-Value	Fold Change	Rank (Top %)	Dataset	#Samples	Reference
**Pancreatic Adenocarcinoma**						
CK2α	0.022	1.74	17%	Iacozb-Donahue	35	[86]
CK2β	0.001	2.2	9%	Logsdon	27	[87]
**Pancreatic Ductal Adenocarcinoma**						
CK2α	0.030	1.5	8%	Grutzmann	25	[88]
CK2α'	0.002	−1.5	5%	Buchholz	38	[89]

Different subtypes of pancreatic cancer are analyzed and p-values, fold changes, and datasets are included.

## Conclusions

It was reported that CK2α protein but not transcripts are deregulated in cancer [22], [27]. In our analysis of Oncomine we found that not only CK2α, but also CK2α', CK2αP, and CK2β transcripts were deregulated in various cancer types (summarized in Table 8). This discrepancy may be due to the fact that these original publications reported data from a limited number of tumor samples (4–8 tumor samples) from two tumor types [22], [27]. However, more recent publications using larger patient samples show that CK2α transcripts are upregulated in lung and colon cancers [34], [39]. Together with our study, these data suggest that deregulated CK2 gene transcript expression may be a mechanism underlying the increase in CK2 activity and protein levels detected in human tumors. However, posttranscriptional and posttranslational mechanisms may also be involved. In general, there is little data on the regulation of CK2 gene expression, and presumably both genetic and epigenetic mechanism may be involved. A number of studies show that regulation of CK2 transcription happens at the promoter level for CK2α and CK2β [43], [44], [45]. However, there is no data on whether CK2 genes themselves are controlled by epigenetic mechanisms. Still, since CK2 controls epigenetic regulators [46], [47], [48], there is a possibility that CK2 could control itself though epigenetic regulation. Nonetheless, there is no data on whether altered CK2 expression in cancer is due to genetic or epigenetic mechanisms. Identifying the genetic and epigenetic mechanisms that control CK2 expression will help understand the role of CK2 in biological processes and in disease.

**Table 8:** Overall trends of CK2 mRNA expression levels in the 6 types of cancers.

	Lung	Breast	Colorectal	Ovarian	Prostate	Pancreatic
CK2α	Up	Up	Up	Up	Up	Up
CK2α'	Up	Down	Up	Down	Up	Down
CK2αP	Up	Up	Up	N/A	N/A	N/A
CK2β	Up/down	Up	Up	Up	Up	Up

Our data indicates that deregulated CK2 expression is an important factor during tumorigenesis. In our Oncomine analysis, the relative fold change in the expression of CK2 genes differs between tumors. In addition, we found a strong correlation between particular CK2 gene expression and certain subtypes of cancer. For example, CK2α' was found underexpressed in breast, ovarian and pancreatic cancers. In addition, while both ductal and lobular breast carcinoma underexpressed CK2α', ductal but not lobular breast carcinoma had upregulation of CK2α. Likewise, the CK2β transcripts were upregulated in both ductal and lobular breast carcinoma when they became invasive. This suggests that CK2β may be required to progress into invasive cancer. Another example is the lack of overexpression of CK2αP in non-neoplastic colon adenomas compared to its overexpression in all three of the colorectal adenocarcinomas.

In contrast with this study on transcript levels, we know relatively little about the expression of CK2 proteins in human cancer and its role in tumorigenesis. A number of publications study CK2 activity and protein levels in a few samples from selected human tumors. As discussed by Tawfic [22], increases on CK2 in cancers based on activity levels alone have to be considered carefully while studies of both protein levels and activity are better indicators of CK2 increases in cancers. In line with our findings, different fold change on the expression of CK2α and CK2β genes is found in kidney tumors [49]. Importantly, in head and neck tumors the level of CK2 correlated with tumor grade and stage [50]. However, a thorough analysis of the specific localization of CK2 transcripts and/or protein is needed to understand the role of CK2 in cancer. In this regard, inmunological studies show CK2 located in the leading edge of the tumor in heterotransplanted human colon tumors in mice [51]. Intriguingly, expression of nuclear CK2α protein was higher in colorectal adenocarcinomas than in adenomas compared to normal tissues, which suggests that CK2α's subcellular localization maybe involved in progression from adenomas to malignant tumors [52].

Our analysis of Kaplan-Meier Plotter also found correlations between CK2 gene deregulation and survival rates (Table 9). Overall, having higher levels of CK2 gene expression led to lower survival amongst patients with lung, breast, and ovarian cancer. Similarly, in head and neck tumors the level of CK2 also correlated clinical outcome [50]. However, in lung adenocarcinoma, displaying a higher level of CK2α' and CK2αP correlates with higher survival rates.

**Table 9:** Overall Survivability as an effect of High Gene Expression Levels.

High levels of Expression:	Lung Adenocarcinoma	Breast Cancer	Ovarian Cancer
CK2α	Not significant	Lower survival	Lower survival
CK2α'	Higher survival	Lower survival	Not Significant
CK2αP	Higher survival	Lower survival	Lower survival
CK2β	Lower survival	Lower survival	Not significant

CK2 is an emerging key target in cancer therapy by impacting not only cell growth and proliferation but also anti-apoptotic activity in malignant cells. Indeed, CK2 inhibitors are being tested for their effectiveness in cancer treatment [11], [27], [53], [54]. In addition, CK2 inhibitors are also studied in conjunction with other antitumor agents such as Melphalan and Imatinib [53], [55]. The database mining findings here greatly support the use of CK2-targeted therapies to treat CK2 upregulation in cancer. For example, CK2α,α' and β genes were found significantly deregulated in colorectal adenomas and adenocarcinomas (this study and [39], [52]), therefore CK2 inhibitors could be used to prevent both the start and progression of colon cancer. Our data indicates that, for some types of cancers, a CK2β-dependent CK2 targeted therapy may be more effective. This is the case of breast cancers, where CK2β overexpression correlated with invasive types of breast carcinoma. This property may be exploited to design more effective CK2 inhibitors. In this regard, compounds such as the inhibitor MNA that inhibits more efficiently CK2 holoenzyme (IC50 = 0.3 µM) than monomeric CK2α (IC50 = 2.8 µm) could be an effective CK2β-dependent CK2 targeted therapy [56], [57]. Our data also indicates that for some cancers such as lung adenocarcinoma, the protective effect in patient survival of CK2α' may be counteracted by CK2 inhibitory drugs. In this case, CK2 inhibitors that target specifically CK2α may not affect the protective role of CK2α'.

The importance of our analysis on CK2 gene transcript expression on cancer development, and on cancer patient diagnosis, treatment and survival needs to be further evaluated. First, the correlation between transcript and protein upregulation for different tumor types needs to be researched, as it is protein activity that will be the target of therapy. Second, determining the specific localization of CK2 proteins and transcripts changes in tumor tissues. Third, additional research on the *in vitro* and *in vivo* tumorigenic potential the understudied CK2 genes (CK2α', CK2β and CK2αP). Fourth, study the effect on tumorigenesis of different fold changes in the expression of CK2 genes, in particular incorporating the knowledge on CK2 protein localization in tumors for *in vivo* studies. Fifth, further research of ovarian, prostate and pancreatic cancer as they had a comparatively small number of samples per dataset, and also analyzing other types of cancer. Sixth, additional research on the impact on patient survival of changes in the CK2 genes in additional cancer types. Seventh, adjusting CK2 therapy development to CK2 cancer-specific expression. All together, these studies could lead to cancer diagnostic and predictive tools, and help develop more effective and specific cancer treatments.
