# Correction: Mining CK2 in Cancer

**DOI:** 10.1371/journal.pone.0120224

**Published:** 2015-03-20

**Authors:** The PLOS ONE Staff

The following information is missing from the Funding section.

This work was supported with funding from the National Institutes of General Medical Sciences (NIGMS); 1R01GM098367. This project was supported by a Boston University’s Undergraduate Research Opportunities Program (UROP) Student Research Award (to CEO).
